# Increase in the Tibial Slope in Unicondylar Knee Replacement: Analysis of the Effect on the Kinematics and Ligaments in a Weight-Bearing Finite Element Model

**DOI:** 10.1155/2018/8743604

**Published:** 2018-07-05

**Authors:** Patrick Weber, Matthias Woiczinski, Arnd Steinbrück, Florian Schmidutz, Thomas Niethammer, Christian Schröder, Volkmar Jansson, Peter E. Müller

**Affiliations:** ^1^Department of Orthopedic Surgery, Physical Medicine and Rehabilitation, University Hospital, Ludwig-Maximilians-University (LMU), Campus Großhadern, Marchioninistr. 15, 81377 Munich, Germany; ^2^BG Trauma Centre Tübingen, University of Tübingen, Schnarrenbergstrasse 95, 72076 Tübingen, Germany

## Abstract

**Introduction:**

Unicompartmental arthroplasty (UKA) of the knee in patients with isolated medial osteoarthritis yields adequate results; however, the survival rate is inferior to that of total knee arthroplasty (TKA). A key factor in the longevity of the implant is the positioning; however, the optimal tibial slope in UKA has not been determined. The aim of this study was to establish a finite element (FE) model and investigate the effect of the tibial slope on the strain of the ligaments, kinematics, inlay movement, and load in the nonreplaced patellofemoral compartment in a medial mobile bearing UKA.

**Materials and Methods:**

An FE model of a leg was established with a virtual UKA implantation with three different tibial slopes (0°, 5°, and 10°). Subsequently, the knee was flexed from 14–73°. In addition, the ground reaction force and the muscles were simulated.

**Results:**

With a higher tibial slope, there was more external rotation of the tibia. An increased tibial slope provided a lateral shift of the patella in the trochlear groove and a more anterior position of the inlay. The ligament strains were also changed, specifically, the anterior portion of the medial collateral ligament and the posterior cruciate ligament (PCL).

**Discussion:**

This study established the first model of a quasidynamic mobile bearing UKA in a leg under weight-bearing conditions. With an increasing tibial slope, there was a higher external rotation of the tibia that created different femorotibial and retropatellar kinematics and different strains in the ligaments. This knowledge adds important information for the optimal tibial slope that has to be determined individually depending on the patient's preoperative kinematics, desired postoperative kinematics, ligament status, and location of the retropatellar chondral damage.

## 1. Introduction

Unicompartmental knee arthroplasty (UKA) produces adequate results in patients with osteoarthritis of the medial compartment of the knee. The developer of the medial Oxford® knee (Biomet, Bridgeton, GB), which is a common UKA implant reported a 20-year survival of 91 % [1]. Conversely, the Swedish knee arthroplasty register reported that the revision rate of UKA was higher than that of total knee arthroplasty (TKA). This higher revision rate may also be due to the fact that UKA is “easier” to revise than TKA [2]. The data from the National Joint Registry confirmed this finding, and in Sweden, the number of UKA procedures has diminished during recent years [3, 4]. This development is surprising as UKA has many advantages over TKA. A UKA can be implanted using minimally invasive techniques: rehabilitation is quicker, and the kinematics are similar to those of the physiological knee [5–8]. Registry Data showed that the Oxford knee score is higher in patients with UKA compared to TKA [2]. Further research is required to determine the factors that improve the longevity of UKA so that it equals that of TKA. The positioning of the implant is a critical factor in the longevity; however, an optimal value for the tibial slope has not been defined. Various authors and manufacturers have recommended an optimal value for the tibial slope, and the range is up to 20° [9]. The tibial slope had a considerable effect on wear [10, 11]. The effects of the different tibial slopes on the ligaments, kinematics of the patella, kinematics of the tibiofemoral joint, and inlay movements have not been analyzed.

To study the knee kinematics, different methods have been developed. Knee cadaver studies are often used; however, they are complex, expensive, and time intensive [12]. Experiments with different implant positions in the same human specimen are complex and sometimes not feasible. Computer simulations with FE models are an alternative as they are efficient and a common method used to study knee biomechanics [13–16]. A weight-bearing FE model was developed and validated for a total knee replacement on a full leg [17].

The aim of this study was to develop a weight-bearing FE model for UKA and to analyze the effect of different tibial slopes on the strain of the ligaments, kinematics, inlay movement, and load in the nonreplaced patellofemoral compartment. The hypothesis was that the tibial slope had a considerable effect on the kinematics of the knee.

## 2. Materials and Methods

### 2.1. FE Model

For the numerical simulation of UKA, a validated knee model of TKA was used and is briefly described below [17].

The in silico model of a left knee with UKA was calculated in Ansys V14 (Ansys, Inc., Canonsburg, PA, USA). The geometrical representation of the knee was generated with the software package Amira (Visage Imaging GmbH, Berlin, Germany) from an MRI Dataset (Siemens, Avanton, 1.5 Tesla) with a 1-mm separation distance in the sagittal, coronal, and transversal plane. The control subject (age 28, weight 80 kg, and height 173 cm) had no pathologic conditions or previous surgical intervention on the lower extremity.

A mobile bearing UKA (Univation, Aesculap Orthopaedics, Tuttlingen, Germany) was implanted virtually and was positioned according to the recommendations of the manufacturer. The positions of the femoral and tibial implant in the AP view were parallel to the mechanical axis of the knee for the femur and 90° to the mechanical axis for the tibia. The flexion of the femoral implant was 5° in relation to the posterior lengthwise axis of the femur. The size of the femoral implant was chosen so that the posterior leg of the prosthesis did not project over the posterior edge of the condyle. The tibial slope was varied. The size was chosen to match to the proximal medial tibia. The femur F2, tibia T3 and inlay 2 sizes were chosen to provide an adequate fit. The inlay height was 7 mm (for femur F2). The position and sizing of the prosthesis were controlled by an experienced orthopedic surgeon (PW).

The linear elastic material properties were defined for the deformable prosthesis components and cartilage tissue [17, 18]. The meniscus was simulated with orthotropic behavior [18].

The ligaments were represented with spring elements in a bundle technique. Therefore, prestrains and stiffness were assigned in a previous FE model (Table 1) [19]. In contrast to the TKA numerical model, the quadriceps tendon was not included, and therefore simulation was stopped at 75° of flexion before the tendon could become wrapped [Hehne 1990].

The cartilage of the tibia, the patella, and the femur and the meniscus and inlay were meshed using tetrahedral volume meshes. The femur and tibia component of the UKA procedure were rigid bodies, and therefore, only the surfaces were meshed. The FE mesh used in the numerical simulation consisted of 10-node-tetrahedral elements for the volume mesh and 6-node-triangular elements for the surface mesh with a mesh size of approximately 1.4 mm. Mesh size was based on the convergence analysis which was conducted in the validation study of the general simulation model [16]. The final mesh had approximately 50000 nodes and a solving time of approximately 15 h.

Frictional contacts between the different structures were established using an augmented Lagrange formulation and a contact specific frictional coefficient (*μ* = 0.002 between the patella and femur, the femur and tibia, and the cartilage and meniscus; *μ* = 0.08 for the inlay and femur and the inlay and tibia) [20, 21]. The fixation between the meniscus and tibia cartilage was simulated with a bonded contact. The femoral and tibial component of the prosthesis were fixed with a nondeformable contact formulation to the bone geometry owing to the rigid body assumption.

The boundary conditions of the FE model were based on an experimental knee rig that was used for the validation of the TKA model [22]. To create the FE model with 75° of flexion, 85 load steps were added to the simulation, and the femoral head moved during the simulation in the direction of the ankle. The muscles, i.e., the vastus lateralis and vastus medialis, were simulated with a constant load of 20 N, and the hamstring muscles (the biceps femoris and semitendinosus) were simulated with a constant load of 2 × 10 N during the flexion cycle. To represent a weight-bearing knee with a 50 N ground reaction force, the vastus intermedius was adapted in each load step based on the ground reaction force measured at the distal tibia. A restarting algorithm was established within the FE model to restart the model automatically after a load step in the ground reaction force was not between 50 N and 55 N [12].

Three different numerical models with different slope conditions (0°, 5°, and 10° posterior slope) were generated and calculated. All boundaries were equal in these models. The slope of the tibial baseplate for the 0° slope model was measured in relation to the tibial proximal anatomical axis (TPAA) [23]. The slope modification rotation axis was 2.2 cm from the posterior edge and 2.5 cm from the anterior edge of the tibial baseplate. The height of the tibial baseplate was adapted to have less than 0.25° differences in the varus-valgus position of the tibia at the beginning of the simulation to eliminate an influence of the different ligament strains. The 5° model and the 10° model had a 0.8 mm less resection on the tibia bone than that of the 0° slope model.

To analyze the effect of the different slope conditions, possible influenced parameters were chosen: the patellofemoral kinematics (patella tilt, flexion, shift, and rotation), forces in the ligaments (posterior cruciate ligament anterior (PCLa) and posterior part (PCLp), lateral collateral ligament (LCL), medial collateral ligament anterior (MCLa), medial collateral ligament superficial (MCLs), medial collateral ligament oblique (MCLo)), and the inlay movement (the position of the inlay on the tibial baseplate).

## 3. Results

### 3.1. Femorotibial Kinematics

**Figure 1:**
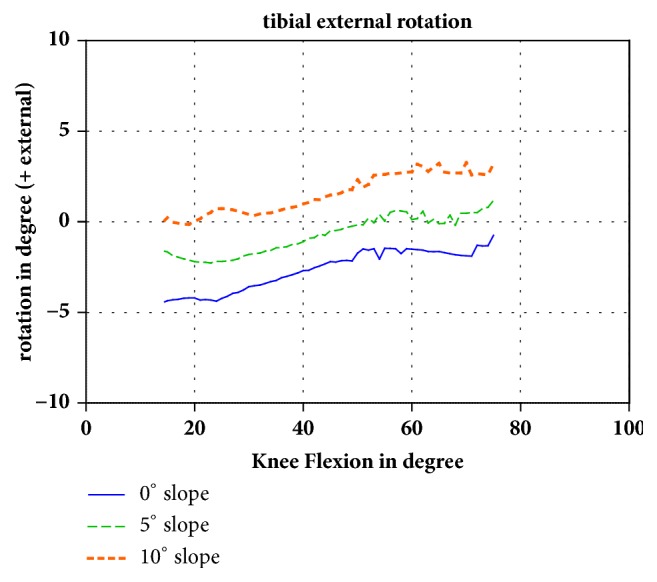
Tibial external rotation in relation to the femoral epicondyles during flexion of the knee (positive value: external rotation). The different slopes are shown in different colors.

The femorotibial kinematics showed a tibial external rotation in relation to the femoral epicondyles during flexion in all numerical models independent of the tibial slope (Figure 1). When the tibial slope increased from 5° to 10°, the tibial external rotation increased. The difference between the 0° and 10° tibial slope groups was a 4.5° external rotation at 14.5° of flexion and 3.9° external rotation at 75° of flexion.

**Figure 2:**
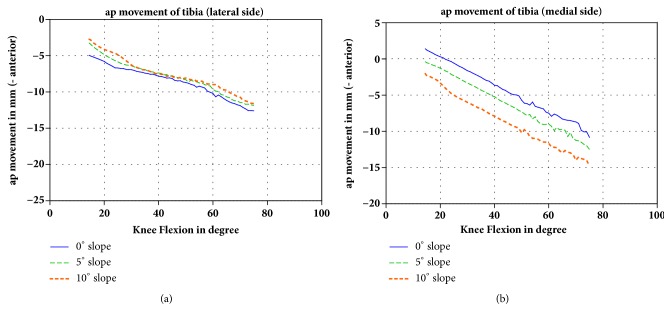
(a) AP movement (translation) of the lateral tibial side in mm in relation to the femoral epicondyles during flexion of the knee (negative value: anterior movement) and (b) AP movement of the medial side. The different slopes are shown in different colors.

Increasing the tibial slope had nearly no effect on the anterioposterior movement (translation) of the tibia on the lateral side during flexion of the knee (Figure 2(a)). However, on the medial side, a higher tibial slope produced an anterior translation of the tibia in relation to the femoral epicondyles (Figure 2(b)). The medial side of the tibia was 3.4 mm and 4.0 mm, respectively, more anterior in the 10° slope model than that of the 0° slope model at 14.5° and 75°, resp., of flexion.

### 3.2. Patellofemoral Kinematics

**Figure 3:**
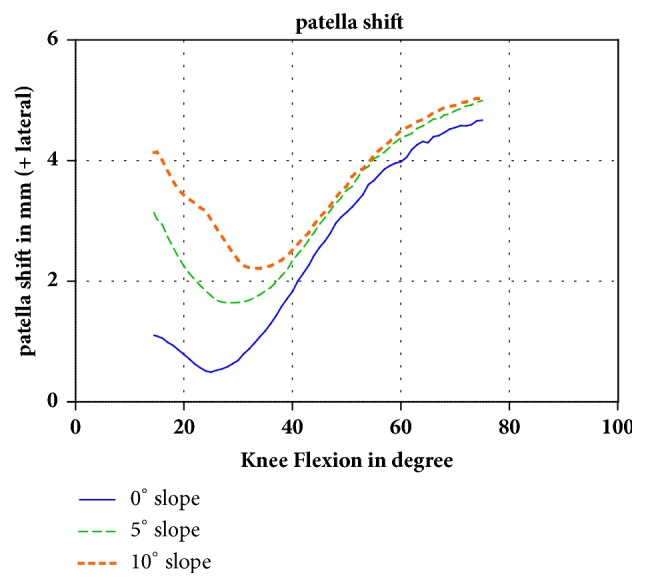
Changes of the patellofemoral kinematics with increasing tibial slope. In early flexion, there was a pronounced lateral shift of the patella in the higher tibial slope groups. With increasing flexion, the difference was reduced.

For the patellofemoral kinematics, only the mediolateral position (shift) of the patella in relation to the femur was influenced by the tibial slope. At 14.5° of flexion, the patella was shifted 3.0 mm more laterally in the 10° slope model than that of the 0° slope model. This difference was reduced at 40° of flexion. At 75° of flexion, there was no more difference between the 0° and 5° tibial slope, and the difference between the 0° and 10° slope was reduced to 0.4 mm (Figure 3). The flexion of the patella in the sagittal plane, the rotation of the patella in the frontal plane, and the tilt of the patella showed no major changes.

### 3.3. Ligament Strains

**Figure 4:**
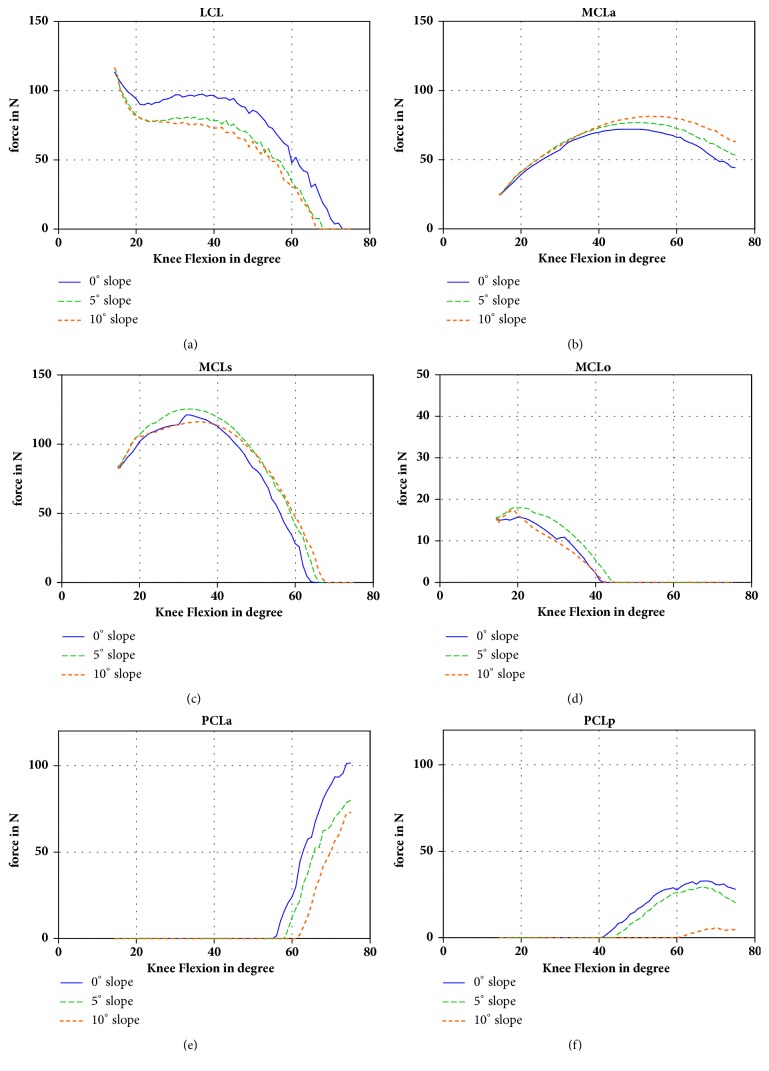
Forces in the different ligaments during flexion of the knee (LCL, MCLa, MCLo, MCLs, PCLa, and PCLp).

The ligament forces are shown in Figures 4(a)–4(f). The force in the MCLa was changed owing to the slope modification. With a 10° slope the strain increased 19 N (63 N at a 0° slope, 44 N at a 10° slope) at 75° of flexion. A similar difference was observed for the MCLs. For the oblique portion, this difference was approximately zero. Conversely, the LCL was 97 N with the 0° slope and 73 N with the 10° slope at 40° of flexion. When the tibial slope increased, the force in the PCL was reduced. The strain of PCLa was 102 N at the 0° slope and 72 N at the 10° slope at 75° of knee flexion. For the PCLp, it was 28 N for the 0° slope model and 4 N for the 10° slope model at 75° of flexion.

### 3.4. Inlay Movement

The position of the inlay in relation to the tibial baseplate shifted anteriorly with an increased slope. At 14.5° flexion the distance of the inlay to the posterior edge of the tibial baseplate was 17.1 mm for the 0° slope, 18.7 mm for the 5° slope, and 20 mm for the 10° slope.

**Figure 5:**
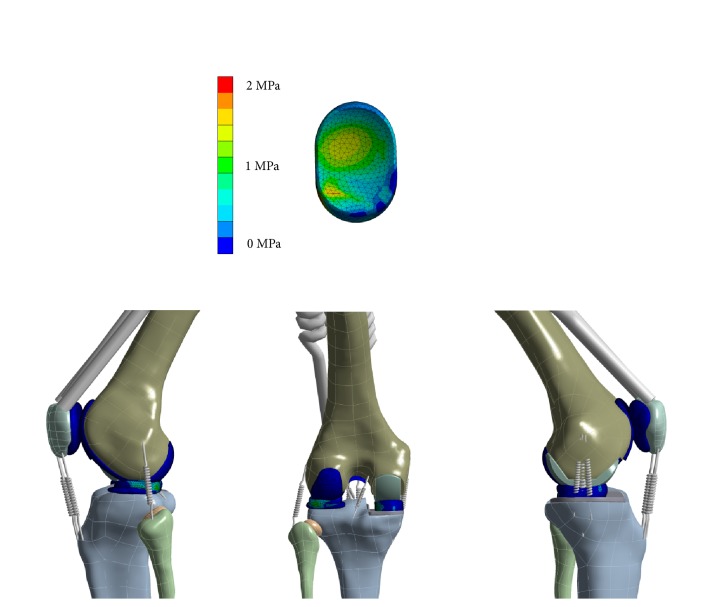
Overview of the von Mises stress in the FE simulation at 40 degree of flexion and stress distribution (von Mises) of the PE inlay.

An overview of the FE model, including a detailed view of the von Mises stress of the PE inlay is shown in Figure 5.

## 4. Discussion

This study established a quasidynamic FE model of UKA in an entire leg. The model was quasidynamic because it studied the kinematics of the entire leg between 10 and 75° of flexion. Previous models were static FE models or did not consider the entire leg [24–27]. The observed strains of the ligaments were similar to those of the TKA model of Steinbrück et al. [12], a model that was validated in a previous study [17]. Therefore, the established model was assumed to be reliable.

The tibiofemoral kinematic analysis showed an anterior translation of the tibia during flexion; therefore, the model showed a physiological roll-back. With an increasing tibial slope, the roll-back was more pronounced on the medial side than on the lateral side. The anterior translation of the tibia on the medial side was because the higher slope leads to a “falling down” of the femoral condyle on the tibial slope and thus a posterior translation of the femur on the medial side. Because there was less movement on the lateral side, the external rotation of the tibia increased 4° with an increased tibial slope.

**Figure 6:**
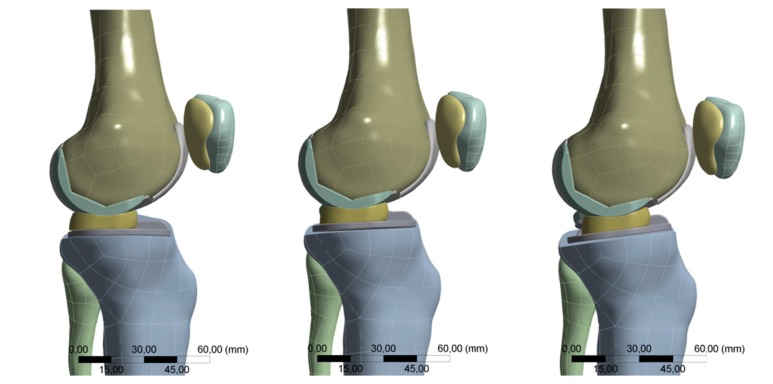
Position of the inlay on the left side with a 0° slope and on the right side with a 10° slope. With an increasing slope, the mobile bearing inlay shifted ventrally because of the congruent spherical form of the femur.

The observed anterior translation of the inlay is shown in Figure 6.

The lateral shift of the patella with an increasing tibial slope was because of the external rotation of the tibia and thus the lateralization of the tibial tubercle. However, the patella in the 5° and 10° slope models shifted back to the position of the 0° slope with an increasing flexion of more than 35°. Our hypothesis to explain this observation is that the patella is captured by the femoral trochlear groove, which is physiological from 30° flexion on [28]. The lateral shift of the patella is important for patients with retropatellar osteoarthritis scheduled for UKA [29]. Patients with osteoarthritis of the medial facet of the patella should have the UKA implanted with an increased tibial slope as this will create a lateral shift of the patella and reduce the strain on the medial side of the patella (Woiczinski, Steinbrück, submitted). For patients with osteoarthritis of the lateral facet of the patella, UKA should be implanted with a reduced tibial slope.

The anterior translation of the tibia on the medial side with an increased tibial slope created an increased strain of the ligaments on the medial side, specifically the MCLa, which contained more strain in flexion [12]. On the lateral side, a slight decrease of the strain was observed. This was owing to the external rotation of the tibia.

The reduction of the strain in the PCL with an increasing tibial slope was also because of the external rotation. The external rotation of the tibial attachment of the PCL approached the femoral attachment of the PCL and reduced the strain.

An optimal value for the tibial slope had not been defined in UKA [9]. In a retrospective analysis of UKA failures, Aleto et al. showed that, for 15 out of 32 cases, the failures were caused by the collapse of the tibial component. Knees with an anterior collapse showed a reduced mean tibial slope of 4.8°, while knees with a dorsal collapse showed a higher tibial slope of 12.8°. The authors recommended a tibial slope of 7° [30]. In a finite element analysis, Sawatari et al. showed a reduced load in the cancellous bone stresses with a slope of 0° [31]. However, the study was a static computer model, and the results were not confirmed experimentally. When the influence on the ligaments in UKA was considered, Hernigou and Deschamps showed, in a clinical study, that a high tibial slope > 13° frequently ruptured the anterior cruciate ligament [32]. In a recent analysis, an implantation with a 10° tibial slope increased the von Mises stress in the bone [25]. A previous study showed that a higher tibial slope reduced the wear in an in vitro experiment [11]. The results of all these studies did not define an optimal value for the tibial slope in all the patients as the results were contradictory. Based on the results of this and all the other studies, an individual tibial slope is required for each patient. A patient with a weak PCL should be implanted with an increased tibial slope, and a patient with a weak ACL should be implanted with a reduced tibial slope based on the results of Hernigou et al. [32]. In addition, individual positioning of the tibial slope should be considered in patients with retropatellar osteoarthritis (OA) and scheduled for UKA. Patients with OA on the lateral facet of the patella should have less tibial slope, and a higher tibial slope should be used for patients with OA on the medial facet of the patella.

*Limitations.* There are limitations that should be considered when transferring the numerical simulation data to the patient. The validation model with a TKA showed good results with 15 specimens experimentally tested in a knee rig [16]; however the geometric model was represented by only one patient aged 28 years and weight 80 kg which may have influenced the results and should be taken into account when transferring the results to patients older or heavier.

First, this experimental study relies on the problem that not all in vivo situations can be restored when a biomechanical setup is developed. In a similar study, weight bearing was simulated even when the 50-N ground reaction force was not a realistic patient weight. However it was shown that the most influence is given by weight bearing versus passive motion and therefore transferable results were generated [33]. Furthermore, in this study, the hamstring and vastus medialis and lateralis were simulated with a constant load that may have influenced the study results.

A further limitation of this model was discussed in a previous paper [12]. Higher flexion grades of the knee were not analyzed as the simulation was manually stopped at 73° of flexion to avoid contact of the quadriceps tendon to the femur because contact was not included in the numerical model. The analyzed 70° of flexion is specific to normal walking [34], and the results may be transmitted to daily activity. However the results may not be transmitted to squatting.

The ligaments were not fully reconstructed as meshed bodies, and the material properties used in the simulation were linear elastic. The ligaments were reconstructed with spring elements in a bundle technique, which is a common technique in FE studies. Adequate agreement with the experimental setups [19, 35] was shown.

Further the bone-implant interface was simulated with a nondeformable contact and no cement mantle which has to be taken into account when comparing the results with the in vivo situation.

The last limitation was that the ACL was not analyzed. The strain in the ACL is however approximately zero in the analyzed range of motion of 70° of flexion.

This study was performed on a mobile bearing UKA; the kinematics of a fixed bearing UKA would probably be different and should be investigated in the future.

## 5. Conclusion

This study established the first model of a quasidynamic mobile bearing UKA in an entire leg, including a simulation of the body weight. In this FE model an increased tibial slope created a higher external rotation of the tibia and thus different femorotibial and retropatellar kinematics and strains in the ligaments. It could be that the in vivo kinematics are not exactly the same as in this model; they should however be similar. So these different kinematics should be considered individually when implanting the UKA in a patient. The optimal tibial slope is different in each patient and should be determined individually depending on the preoperative kinematics, postoperative desired kinematics, ligament status, and location of the retropatellar chondral damage.

## Figures and Tables

**Table 1:** Overview of the material properties used in the FE simulation.

**Deformable bodies (linear elastic)**					
Structure	Young's modulus (MPa)	Poisson ratio	Structure	Young's modulus (MPa)	Poisson ratio

Femoral component	210,000	0.3	Patella Cartilage	5.0	0.4
Inlay (UHMWPE)	312.5	0.46	Tibial Component	210,000	0.3

**Ligaments (stiffness)**					

Structure	Initial strain	Stiffness (N/mm)	Structure	Initial strain	Stiffness (N/mm)

PCLa	-0.10	31.25	MCLa	0.02	27.9
PCLp	-0.02	15.0	MCLo	0.02	21.1
LCL	0.02	91.3	MCLs	0.02	72.2
ACLa	0.02	108.0	ACLp	0.02	108.0

## Data Availability

The data used to support the findings of this study are available from the corresponding author upon request.
